# Human parainfluenza virus type 3 expressing the respiratory syncytial virus pre-fusion F protein modified for virion packaging yields protective intranasal vaccine candidates

**DOI:** 10.1371/journal.pone.0228572

**Published:** 2020-02-11

**Authors:** Xueqiao Liu, Bo Liang, Xiang Liu, Emerito Amaro-Carambot, Sonja Surman, Peter D. Kwong, Barney S. Graham, Peter L. Collins, Shirin Munir

**Affiliations:** 1 RNA Viruses Section, Laboratory of Infectious Diseases, National Institute of Allergy and Infectious Diseases, National Institutes of Health, Bethesda, Maryland, United States of America; 2 Vaccine Research Center, National Institute of Allergy and Infectious Diseases, National Institutes of Health, Bethesda, Maryland, United States of America; University of Iowa, UNITED STATES

## Abstract

Human respiratory syncytial virus (RSV) and parainfluenza virus type 3 (HPIV3) are among the most common viral causes of childhood bronchiolitis and pneumonia worldwide, and lack effective antiviral drugs or vaccines. Recombinant (r) HPIV3 was modified to express the RSV fusion (F) glycoprotein, the major RSV neutralization and protective antigen, providing a live intranasal bivalent HPIV3/RSV vaccine candidate. This extends previous studies using a chimeric bovine-human PIV3 vector (rB/HPIV3). One advantage is that rHPIV3 expresses all of the HPIV3 antigens compared to only two for rB/HPIV3. In addition, the use of rHPIV3 as vector should avoid excessive attenuation following addition of the modified RSV F gene, which may occur with rB/HPIV3. To enhance its immunogenicity, RSV F was modified (i) to increase the stability of the prefusion (pre-F) conformation and (ii) by replacement of its transmembrane (TM) and cytoplasmic tail (CT) domains with those of HPIV3 F (H3TMCT) to increase incorporation in the vector virion. RSV F (+/- H3TMCT) was expressed from the first (F/preN) or the second (F/N-P) gene position of rHPIV3. The H3TMCT modification dramatically increased packaging of RSV F into the vector virion and, in hamsters, resulted in significant increases in the titer of high-quality serum RSV-neutralizing antibodies, in addition to the increase conferred by pre-F stabilization. Only F-H3TMCT/preN replication was significantly attenuated in the nasal turbinates by the RSV F insert. F-H3TMCT/preN, F/N-P, and F-H3TMCT/N-P provided complete protection against wt RSV challenge. F-H3TMCT/N-P exhibited the most stable and highest expression of RSV F, providing impetus for its further development.

## Introduction

Human respiratory syncytial virus (RSV) is an enveloped, non-segmented, negative sense RNA virus with a genome of approximately 15.2 kb [1]. It is classified in the genus *Orthopneumovirus* and family *Pneumoviridae* [2]. RSV is the most common cause of viral bronchiolitis and pneumonia in infants and young children and lacks a vaccine or an effective antiviral drug. It is estimated that RSV is associated annually with 34 million lower respiratory tract infections and 4 million hospitalizations [3]. The annual RSV-related death burden in all age groups is 234,000–520,000 worldwide including 66,000–199,000 in children younger than 5 years of age [4].

RSV encodes three virion surface glycoproteins: attachment protein G, small hydrophobic protein SH, and fusion protein F. The F and G proteins are the viral neutralization and major protective antigens, of which F is generally thought to play a greater role in eliciting a protective antibody response. The F protein is a type I integral membrane protein (i.e., anchored near the C-terminus) that mediates fusion of the viral envelope with the cellular plasma membrane or intracellular vesicle membranes for viral entry. RSV F is initially synthesized as an inactive F_0_ precursor that is sequentially cleaved by the intracellular furin protease at two sites (first during synthesis and second concomitant with entry), located 27 amino acids apart, generating a smaller N-terminal F2 fragment, a larger C-terminal F1 fragment, and a small 27-amino-acid intervening fragment. F2 and F1 remain held together by disulfide bonds. Newly-synthesized RSV F has a metastable prefusion (pre-F) conformation. During fusion, and also sometimes spontaneously, pre-F undergoes a major, irreversible conformational change to a more stable post-fusion (post-F) form. The F protein on the surface of RSV particles typically is found in both the pre-F and post-F conformations, with the latter often being predominant [5]. Both pre- and post-F possess RSV neutralization epitopes [6–8]. However, most of the RSV-neutralizing activity in human sera is contributed by antibodies that are specific for epitopes unique to pre-F [6, 8], in particular antigenic site Ø, and are highly effective in RSV neutralization. This suggests that pre-F is the preferred antigenic form for an RSV vaccine. The metastable pre-F conformation can be substantially stabilized by introducing mutations, such as two new cysteine residues S155C and S290C to create a new, stabilizing disulfide bond (called DS), and the hydrophobic cavity-filling mutations S190F and V207L (called Cav1).

Human parainfluenza virus type 3 (HPIV3) is second only to RSV as a viral cause of severe respiratory illness in early childhood worldwide [9]. HPIV3 also lacks a vaccine or antiviral drug. Like RSV, HPIV3 is an enveloped, single stranded, negative sense RNA virus. However, it is substantially divergent from RSV and is classified in the genus *Respirovirus*, family *Paramyxoviridae*. The genome of HPIV3 is 15,462 nucleotides in length and contains six genes in the 3’-5’order of nucleoprotein (N), phosphoprotein (P), matrix protein (M), fusion glycoprotein (F), hemagglutinin-neuraminidase (HN), and large polymerase (L). In addition, the P gene contains a second ORF encoding the nonstructural C protein, and RNA editing of the P gene accesses an additional ORF to express a protein called D of uncertain significance [9].

Previous experimental vaccines based on inactivated RSV or RSV subunit proteins often have been poorly protective and, in RSV-naïve young children, induce an atypical immune response that causes enhanced disease during subsequent natural infection. Markers of enhanced disease also have been reported in experimental animals for inactivated HPIV3 vaccines [10]. However, live RSV and HPIV3 vaccines do not cause disease enhancement, and suitably-attenuated live RSV and HPIV3 vaccines are safe in this population [11].

We have been pursuing a bivalent vaccine approach in which live-attenuated recombinant strains of PIV are used as vectors to express RSV antigen, especially the RSV F protein, from an added gene [12–17]. Much of this work involved an attenuated recombinant vector called rB/HPIV3 that is a chimera of bovine PIV3 (BPIV3) and HPIV3. This chimeric virus consists of BPIV3 in which the genes encoding the surface F and HN glycoproteins were replaced with those of HPIV3, thus providing homologous neutralization antigens against HPIV3. BPIV3 and rB/HPIV3 are highly attenuated in humans due to host range restriction [18, 19]. We have shown that RSV F stabilized in its pre-F conformation and expressed from a rB/HPIV3 vector is very immunogenic and induces serum antibodies that are strongly neutralizing *in vitro* even in the absence of added complement, which we call “high quality” antibodies. In contrast, high quality antibodies were not efficiently induced by unmodified RSV F. We also previously showed that pre-F immunogenicity can be further enhanced by increasing its incorporation in the vector particles by replacing its transmembrane (TM) and cytoplasmic tail (CT) domains with their counterparts from the vector F protein. These two modifications, namely pre-F stabilization and virion packaging, individually and additively increase the titers of high quality serum RSV-neutralizing antibodies that are important for protection [13–15].

In the present study, we evaluated the use of unmodified rHPIV3 (JS strain) as a vector. This provides a new lineage of vaccine candidates in which all of the viral genes are from HPIV3, and which is less attenuated than rB/HPIV3.

## Results

### Generation of rHPIV3 viruses expressing RSV pre-F with or without H3TMCT

cDNAs encoding the RSV F protein of strain A2 were synthesized with a number of modifications. The encoded protein was modified by inclusion of the “DS” mutations (S155C and S290C) and the “Cav1” mutations (S190F and V207L) that increase the stability of the pre-F conformation (Introduction). The F ORF was codon-optimized for enhanced translation. Two amino acid changes (K66E and Q101P, called the “HEK” mutations) were included that make F identical at the amino acid level (apart from the DS-Cav1 mutations) to an early passage of the A2 strain [20]. A second version of this RSV F construct was made with all of these changes plus the substitution of the RSV F TM and CT domains with those of rHPIV3 F (H3TMCT), in an effort to increase packaging into the rHPIV3 vector particles.

**Fig 1:**
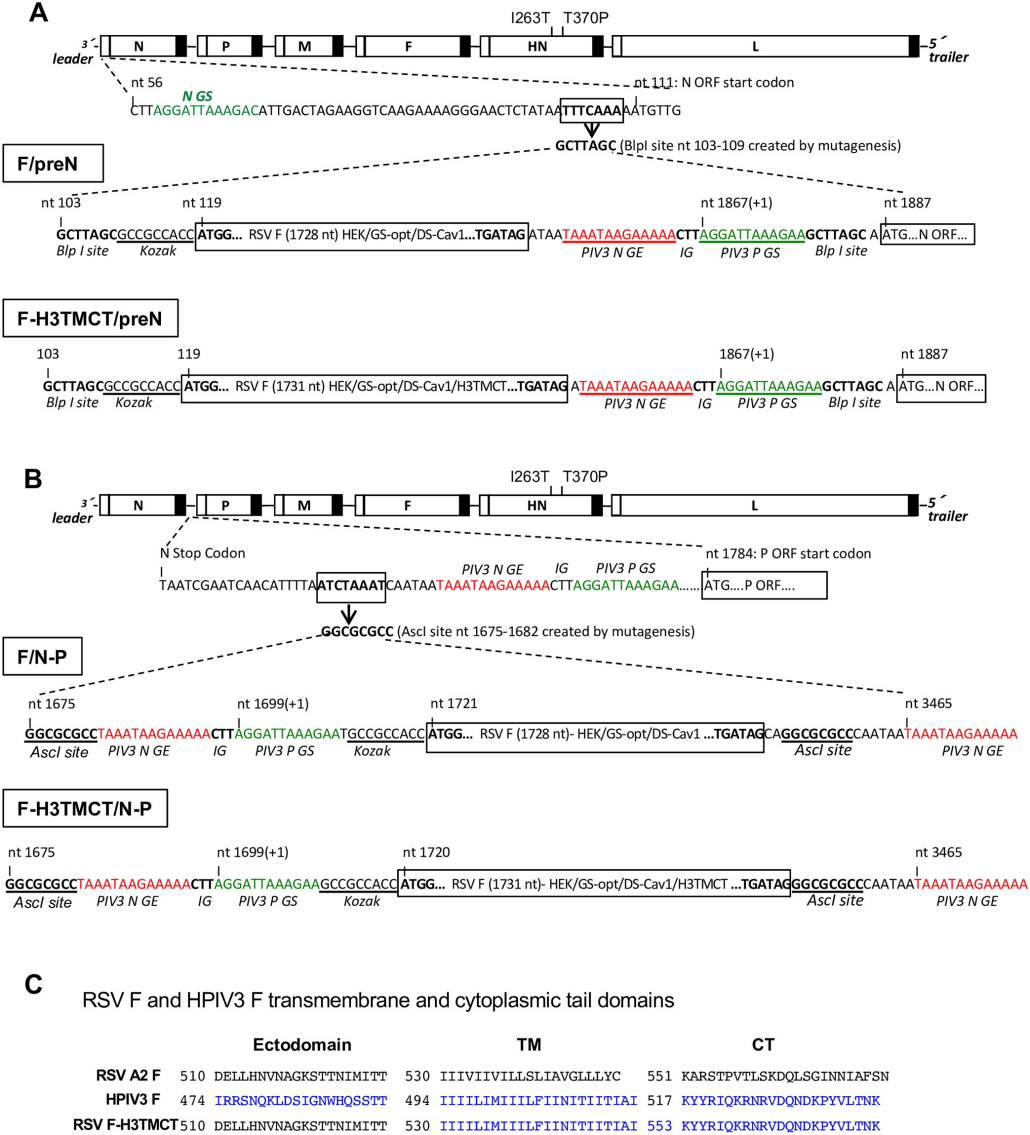
Genome structures of the rHPIV3-RSV-F vectors. The codon-optimized RSV F ORF containing the HEK and DS-Cav1 mutations (see Results) was engineered to contain an optimal translational start site (underlined and labeled “Kozak” [36]) and was flanked by HPIV3 gene-end (GE) and gene-start (GS) transcription signals and the intergenic CTT trinucleotide to allow for expression as a separate mRNA. RSV F was either the full-length protein (constructs F/preN and F/N-P) or a chimeric version in which the transmembrane (TM) and cytoplasmic tail (CT) domains of RSV F were replaced by those of the HPIV3 F protein (constructs F-H3TMCT/preN and F-H3TMCT/N-P). The insert was placed (A) at the first gene position using the *Blp*I site or (B) the second gene position using the *Asc*I site in the wild type (wt) rHPIV3 JS strain vector. The vector contained I263T and T370P amino acid substitutions that restore the wt sequence (see the text). (C) Nucleotide sequences of the TM and CT domains and part of the adjoining ectodomains of RSV strain A2 F protein (top), HPIV3 F protein (middle), and chimeric RSV-F-H3TMCT protein.

The resulting RSV F cDNAs, encoding either the full-length protein or the H3TMCT version, also were designed to be flanked by HPIV3 gene-end (GE) and gene-start (GS) transcription signals and *Blp*I or *Asc*I sites (Fig 1) to allow for insertion into the rHPIV3 antigenome and expression as a separate mRNA. RSV F or RSV F-H3TMCT was inserted at the first (Pre-N, *Blp*I site) (Fig 1A) or the second (N-P, *Asc*I site) (Fig 1B) gene position in a cDNA encoding the antigenomic RNA of wild type (wt) rHPIV3, JS strain. This antigenomic cDNA was the same as previously described [21] except that two amino acid changes that were originally made in the HN protein (T263I and P370T) as markers were changed back to their wt assignments (T and P, respectively). The resulting cDNA constructs encoded the antigenomic RNAs for four viruses, namely F/preN, F-H3TMCT/preN, F/N-P, and F-H3TMCT/N-P (Fig 1; Materials and Methods). The sequences of the TM and CT domains of RSV F, HPIV3 F, and RSV F-H3TMCT are shown in Fig 1C.

### Virus recovery, stability of expression of RSV F protein, and plaque phenotypes

The four constructs were transfected in duplicate into BHK BSR-T7/5 cells that were incubated at 32°C for 48 h, scraped into the medium, and plated onto subconfluent monolayers of LLC-MK2 cells. The cells were incubated at 32°C until cytopathic effects were evident, and the tissue culture medium supernatants were harvested and clarified to provide passage 1 (P1) virus stocks. The stability of expression of RSV F protein was evaluated by a double-staining immunofluorescence plaque assay that simultaneously detects the expression of RSV F protein and several HPIV3 virion proteins. To do so, Vero cells were infected with serial 10-fold dilutions of the P1 stocks, incubated for 6 days under methylcellulose, and immunostained with a mixture of three conformationally-dependent MAbs specific to RSV F [22] (visualized as red) and a polyclonal antiserum against HPIV3 virions (visualized as green). Plaques co-expressing RSV F and rHPIV3 antigens would appear yellow whereas those that had lost detectable expression of RSV F protein would appear green.

Unexpectedly, the P1 stocks of each of the four constructs from this initial transfection exhibited a mixture of two plaque sizes. The majority of plaques for each construct were similar in size (F/N-P and F-H3TMCT/N-P), or smaller (F/preN and F-H3TMCT/preN) than the plaques of wt rHPIV3. The reduced size for F/preN and F-H3TMCT/preN suggested that insertion of RSV F at the pre-N position reduced viral replication and spread. The TMCT modification on its own was not associated with any apparent change in plaque size for either insertion site. For each construct, a minority of plaques had a large-plaque phenotype. Also, a small minority of plaques for each construct were green, indicating a loss of expression of detectable RSV F. This did not correlate with any particular plaque size.

In an effort to isolate partially-cloned, enriched populations, one P1 stock for each of the four constructs was subjected to two rounds of terminal dilution on LLC-MK2 cells. Terminally diluted stocks with low proportions of plaques with the large-plaque phenotype and/or loss of RSV F expression were not obtained for the F/preN and F/N-P constructs, but were provisionally obtained for the F-H3TMCT/preN and F-H3TMCT/N-P constructs. One such stock for each of these latter two constructs was passaged once in LLC-MK2 cells and analyzed by complete genome sequencing. The F-H3TMCT/N-P stock lacked detectable adventitious mutations and was used for further characterization. However, the F-H3TMCT/preN stock was found to contain a missense mutation in L (L1568I) and was not further analyzed. In addition, we performed nucleotide sequence analysis on eight other terminally-diluted viral stocks that had lost RSV F expression or had the large plaque phenotype and results are shown in Table 1. In six instances where expression of RSV F was lost, sequence analysis of the F gene and flanking regions found four instances of an introduced stop codon in the RSV F ORF, one instance of a frameshift, one instance of a single missense mutation in RSV F and one instance of three missense mutations (Table 1, rows 1–4, 7 and 8). The missense mutations presumably would not prevent translation of the RSV F ORF but may have interfered with detection of RSV F protein by the three conformationally-dependent MAbs used for plaque staining through effects on conformation and possible ablation of one or more epitopes and/or reduction in protein stability. Complete genome sequencing of two large-plaque stocks found that one had a Y3H mutation in the HN protein and V242G, N248K, C252W, F277V, and I279M mutations in the L protein (Table 1, row 5), while in the other viral stock no predominant mutation was detected (row 7).

**Table 1:** Mutations detected in examples of viruses showing large plaques and/or loss of RSV F expression.

Virus clone	Construct	Plaque size	RSV F expression detected	RSV F Mutation*	HPIV3 Vector mutation**
D3-F12	F/preN	Normal	no	Y342C	ND
D11-H5	F/preN	Mixed- normal and large	no	E110 → Stop	None found
E1-B4	F/preN	Normal	no	E110 → Stop	ND
G9-E10	F/preN	Normal	no	“A” insertion at nt 5 caused frame shift and E30 → Stop	ND
C5-E3	F-H3TMCT/preN	Large	yes	No	Y3H (HN protein); V242G, N248K, C252W, F277V, I279M (L protein)
A3-D8	F/N-P	Normal	no	None found	ND
C6-F4	F/N-P	Large	no	L321P, Y342H, F352P	None found
G6-E8	F-H3TMCT/N-P	Normal	no	K132 → Stop	ND

One stock of each virus construct, at P1 stage after rescue, was subjected to terminal dilutions in LLC-MK2 cells. Terminally diluted enriched virus clones that showed large plaque phenotype and/or had lost expression of RSV F were sequenced.

* RSV F insert and flanking transcription signals were sequenced for viruses with loss of RSV F expression.

** HPIV3 backbone was sequenced for clones with large plaques.

ND, not determined.

Two additional independent sets of transfections (#2 and 3) were performed. This was done in an effort to obtain stocks of the other three constructs (F/preN, F-H3TMCT/preN, and F/N-P) that have low proportions of plaques with the large-plaque phenotype and/or loss of RSV F expression, and also to see if the generation of these sub-populations was a consistent finding. Transfection #2 comprised 6 replicates each of the F/preN, F-H3TMCT/preN, and F/N-P constructs. Transfection #3 comprised 2, 2, 4, and 6 replicates for F/preN, F-H3TMCT/preN, F/N-P, and F-H3TMCT/N-P, respectively. Surprisingly, transfections #2 and #3 yielded replicate P1 stocks in which most of the plaques expressed RSV F and there were few large-plaque variants.

**Fig 2:**
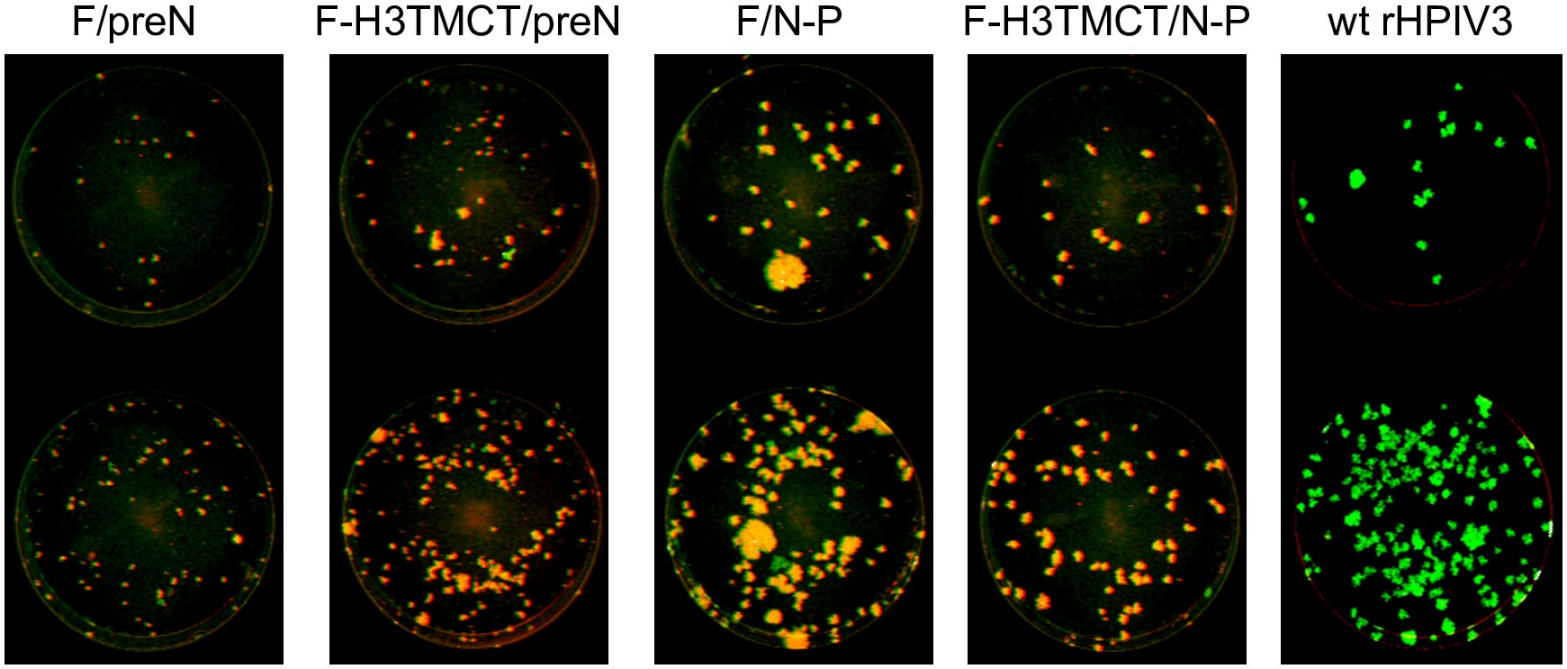
Stability of expression of RSV F protein by rHPIV3-RSV-F vectors evaluated by a double-staining immunofluorescence plaque assay. Vero cells were inoculated with 10-fold serial dilutions of the P4 stock of F-H3TMCT/N-P obtained from terminal dilution from transfection #1 and P2 stocks of F/preN, F-H3TMCT/preN, and F/N-P from transfection #2 (see Results). Cells were infected in duplicate and incubated for 6 days at 32°C under a 0.8% methylcellulose overlay. The cells were fixed and subjected to double-staining immunofluorescence with a rabbit polyclonal hyperimmune serum raised against HPIV3 virions and a mixture of three conformationally-dependent murine anti-RSV F MAbs [22]. Secondary antibodies were infrared dye-labeled donkey anti-rabbit 800-CW and donkey anti-mouse 680-LT. The Odyssey infrared imaging system was used to acquire plaque images. The infrared dyes were pseudo-colored to appear green and red for rHPIV3 and RSV F antigens, respectively. The plaques co-expressing rHPIV3 and RSV F antigens appear yellow. Plaques in which expression of RSV F could not be detected by the three conformationally-dependent MAbs appear green. Representative monolayers are shown.

F/preN, F-H3TMCT/preN, and F/N-P P1 stocks from transfection #2 were passaged once in LLC-MK2 cells to yield P2 stocks and together with the terminally-diluted P4 stock of F-H3TMCT/N-P from transfection #1 described above, these stocks were used for all subsequent experiments. The plaque images of these viruses are shown in Fig 2 alongside wt rHPIV3. This showed that the P2 stock of the F/N-P construct had a relatively higher content of large-plaque variants. The percentages of plaques with loss of expression of RSV F or large-plaque phenotype in these P2 stocks are shown in Fig 9B. Genomes of the rHPIV3-RSV F vectors used for *in vitro* and *in vivo* experiments were sequenced except for the 30 nucleotides at the 3’ genome end for all viruses and 170, 170, 214, and 170 nucleotides for the F/preN, F-H3TMCT/preN, F/N-P, and F-H3TMCT/N-P, respectively at the 5’ genome end. No adventitious mutations were detected.

### Multicycle growth kinetics in Vero cells

**Fig 3:**
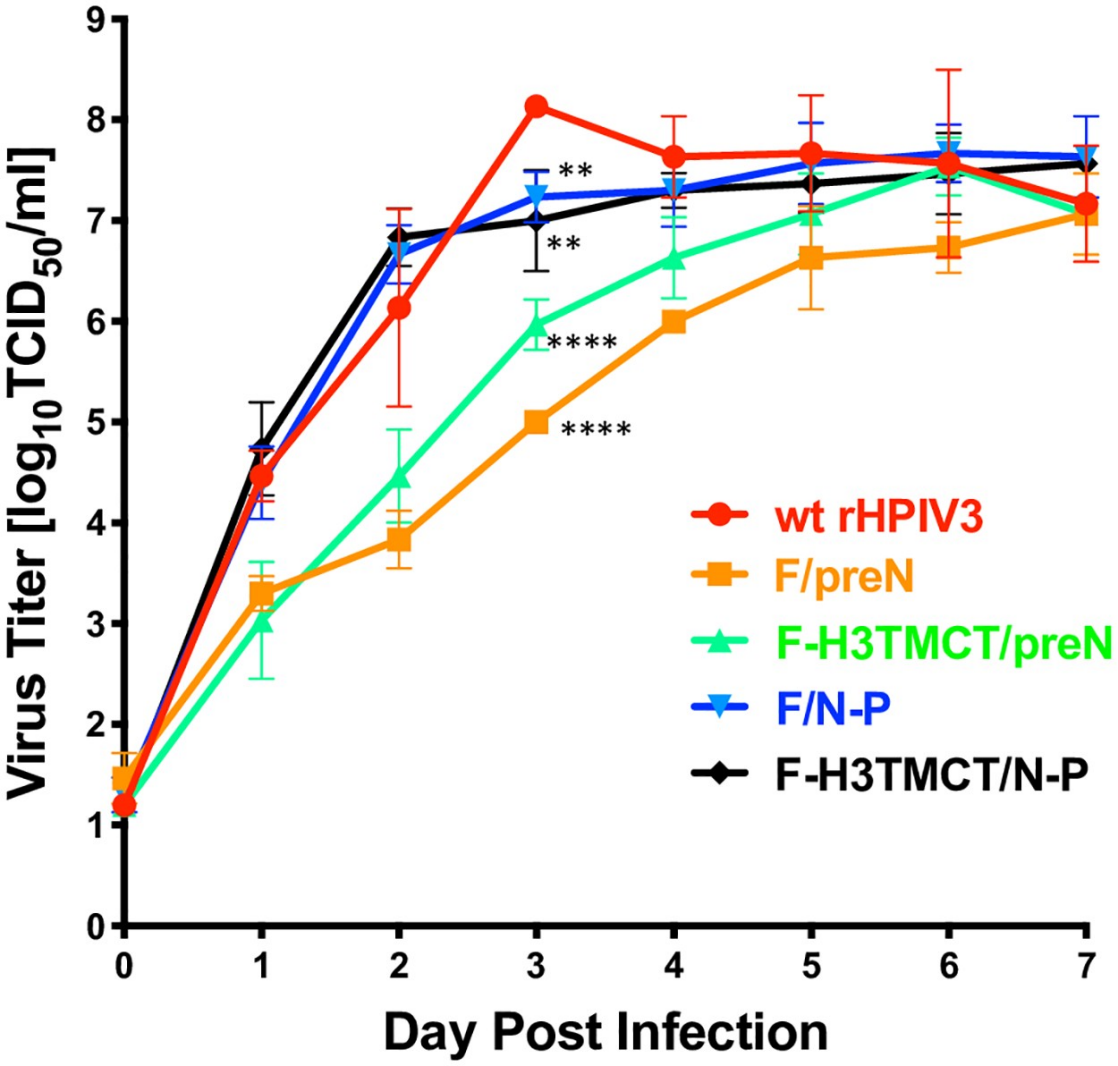
Multistep growth curve of rHPIV3 vectors expressing RSV F. Vero cells were infected in triplicate with an MOI of 0.01 TCID_50_/cell of the indicated viruses. Cells were incubated at 32°C and aliquots of culture medium were collected at 24 h intervals over 7 days and replaced by equal volumes of fresh medium. Samples were flash frozen and at a later time virus titers were determined in parallel by limiting dilution on LLC-MK2 cells using an hemadsorption assay, and are reported as log_10_TCID_50_ per mL. Mean titers are shown with the standard deviations indicated as vertical error bars. The statistical significance of the differences between the vectors expressing RSV F versus wt rHPIV3 empty backbone on day 3 and 7 post-infection was determined by one-way analysis of variance with Tukey’s multiple-comparisons post-test and are indicated by ** (P<0.01) and **** (P<0.0001).

Vero cells were infected with the rHPIV3-RSV-F vectors or with wt rHPIV3 as a reference control. The culture medium of infected Vero cells was sampled at 24 h intervals for 7 days. Virus titers in the supernatant medium samples were determined by terminal dilution on LLC-MK2 cells followed by hemadsorption, and titers were reported as log_10_ 50% tissue culture infective dose per ml (TCID_50_/ml) (Fig 3). Insertion of RSV F into the rHPIV3 genome significantly reduced replication of all viruses on day 3, while insertion into the first gene position (F/preN and F-H3TMCT/preN) reduced replication throughout most of the time course. The significantly slower growth of F/preN and F-H3TMCT/preN as compared to F/N-P and F-H3TMCT/N-P was consistent with their reduced plaque size (Fig 2). Replication of wt rHPIV3 reached peak titer on day 3, while the titers of vectors expressing RSV F continued to increase until day 7, especially for the F/preN and F-H3TMCT/preN viruses. Nevertheless, despite the initial delayed growth, all viruses reached high titers comparable to that of wt rHPIV3 on day 7.

### Expression of RSV F and rHPIV3 proteins in infected cell lysates

**Fig 4:**
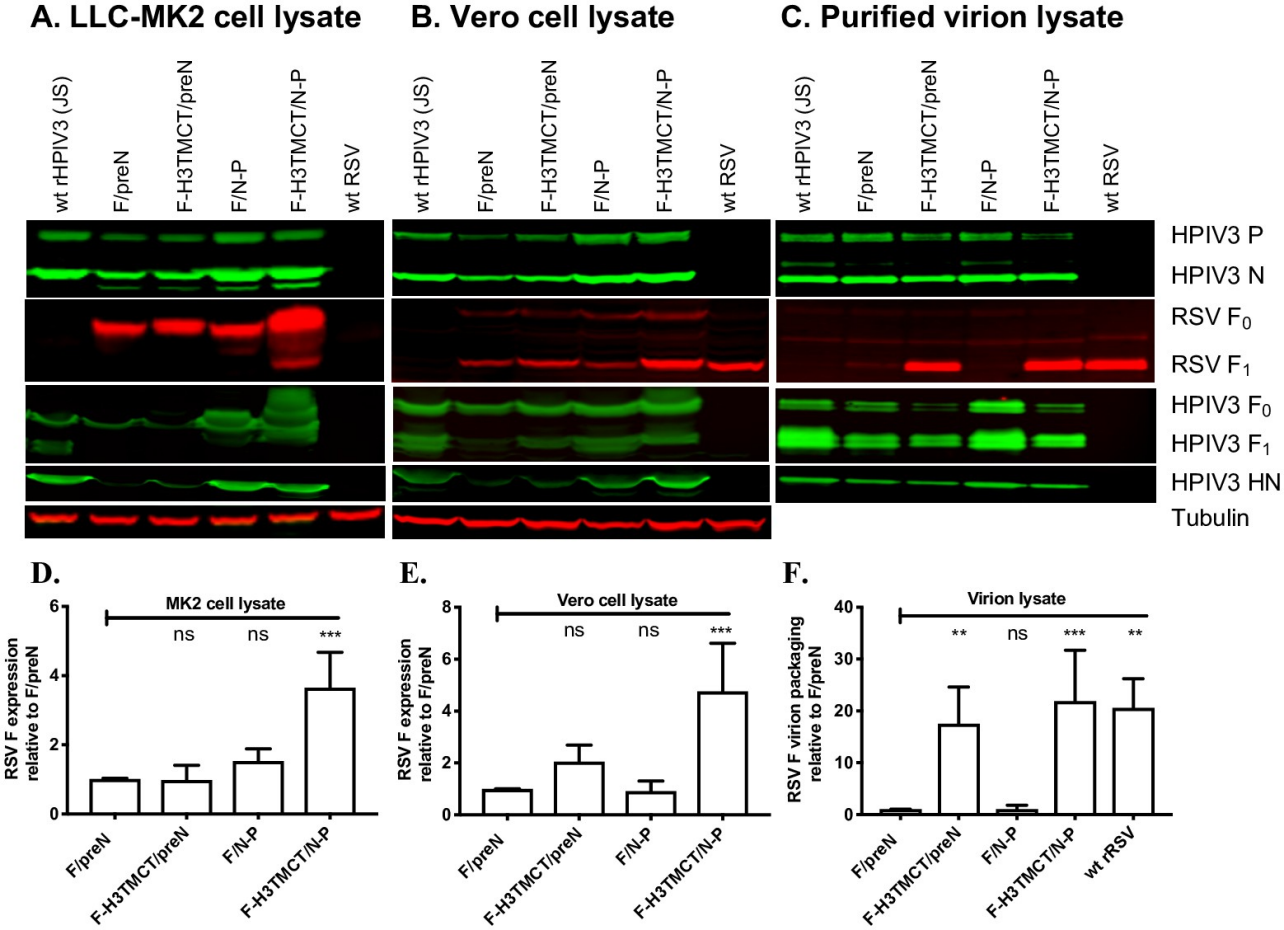
Western blot analysis of infected cell lysates and sucrose gradient-purified virions. (A, B) Analysis of cell-associated proteins. LLC-MK2 (A) or Vero (B) cells were infected with the indicated rHPIV3-RSV-F vectors or wt rHPIV3 at an MOI of 3 TCID_50_/cell or with wt RSV at an MOI of 3 PFU/cell and incubated for 48 h at 32°C, after which cells were lysed in denaturing and reducing sample buffer and analyzed by Western blotting. RSV F was detected with a mouse anti-RSV F MAb; note that expression of F protein by RSV in LLC-MK2 cells (A) was below the level of detection due to inefficient infection by RSV in that cell type. rHPIV3 N and P proteins were detected with rabbit polyclonal hyperimmune serum against HPIV3 virions; rHPIV3 F was detected with a rabbit hyperimmune serum against recombinant purified F ectodomain; and rHPIV3 HN was detected with a hyperimmune rabbit serum raised against an HN peptide. Tubulin was detected on all blots as a loading control using a mouse anti-tubulin MAb. Secondary antibodies are described in Materials and Methods. Representative blots are shown. (C) Analysis of purified virions. LLC-MK2 cells were infected with the indicated rHPIV3-RSV-F vectors or wt rHPIV3 at an MOI of 0.1 TCID_50_/cell, and Vero cells were infected with RSV at an MOI of 0.1 PFU/cell (LLC-MK2 cells were used for rHPIV3, but Vero cells were used for RSV because they are more permissive) and incubated at 32°C. Culture medium supernatants were collected, clarified by low-speed centrifugation, and subjected to sucrose gradient centrifugation to partially purify the virus particles. One μg of total protein from each virus preparation, as measured by BCA assay, was denatured, reduced, and analyzed by Western blotting (as in parts A and B) to quantify packaging of RSV F and rHPIV3 proteins into the vector particles. In panels A-C, blot images are representative of three independent experiments. RSV F protein bands were quantified by densitometry and normalized to tubulin (A and B) or rHPIV3 N protein (C). The values of RSV F from three repeats for each of A, B, and C were plotted in D, E, and F, respectively, as fold change in the amount of RSV F relative to that of the F/preN virus assigned the value of 1.0. Cell-associated RSV F in the LLC-MK2 and Vero cell lysates was predominantly detected as the F_0_ precursor and the larger F_1_ subunit, respectively, and in virions as the F_1_ subunit; these were the forms that were quantified.

To measure the expression of RSV F and rHPIV3 vector proteins in cell culture, LLC-MK2 and Vero cells were infected with the indicated viruses and incubated for 48 h. RSV F and rHPIV3 proteins in cell lysates were analyzed by Western blot analysis (Fig 4A and 4B). In LLC-MK2 cells, insertion of RSV F in the pre-N position slightly reduced rHPIV3 P and N protein expression, compared to the rHPIV3 empty vector, while expression of rHPIV3 F and HN proteins was greatly reduced. Insertion of RSV F at the N-P position had no effect on expression of rHPIV3 P, N, F, or HN. Abundant expression of RSV F (mostly in the form of F_0_) was detected in LLC-MK2 lysates infected with each of the rHPIV3 vectors; F-H3TMCT/N-P expressed a significantly higher amount of RSV F than the other three viruses (Fig 4A and 4D). In contrast, expression of the F protein by wt RSV in LLC-MK2 cells was not readily detected in these experiments due to inefficient infection.

In Vero cells (Fig 4B), F/preN and F-H3TMCT/preN expressed lower amounts of rHPIV3 P, N, F, and HN proteins compared to rHPIV3, whereas F/N-P and F-H3TMCT/N-P showed no effect of the RSV F insertion on vector protein expression: these findings are consistent with the results with LLC-MK2 cells described above. Expression of RSV F protein was detected for all four rHPIV3-RSV-F vectors in Vero cells, but unlike LLC-MK2 cells, RSV F was detected mainly as the F_1_ subunit, indicative of cell-specific differences in the efficiency of cleavage. Consistent with the LLC-MK2 cells, RSV F was expressed in significantly higher amount by F-H3TMCT/N-P as compared to the rest of the candidates (Fig 4B and 4E). Unlike LLC-MK2 cells, expression of F protein by wt RSV was readily detected in Vero cells and was somewhat less abundant than for F-H3TMCT/N-P.

### RSV F with TMCT modification was packaged more efficiently in the rHPIV3 virus particles

In previous studies, wt RSV F protein was not efficiently packaged in the rB/HPIV3 or rHPIV1 virions, with only a trace amount detectable [13, 14]. As noted, the F-H3TMCT/preN and F-H3TMCT/N-P viruses expressed variants of the RSV F protein in which the RSV F TMCT domains were substituted with those of the rHPIV3 F protein in order to facilitate interaction with rHPIV3 proteins involved in virion formation. To detect the incorporation of RSV F protein in rHPIV3 particles, wt rHPIV3, the HPIV3-RSV-F vectors, and wt RSV were sucrose gradient-purified from infected-cell media supernatants, and 1 μg of each virion preparation was subjected to Western blot analysis (Fig 4C). As expected, a very small amount of the full-length RSV F was detected in the F/preN and F/N-P virions whereas the F-H3TMCT/preN and F-H3TMCT/N-P virions appeared to package RSV F bearing the H3TMCT modification with an efficiency comparable to that of wt RSV (based on the equal-weight comparison in Fig 4C). The increase in packaging for F-H3TMCT/preN—and F-H3TMCT/N-P was 17.5- and 21.9-fold compared to F/preN and F/N-P, respectively. Increased packaging of the chimeric RSV F in F-H3TMCT/preN and F-H3TMCT/N-P virions was associated with reduced packaging of the vector F protein, likely due to competition for packaging between their identical TMCT domains. Packaging of the vector HN protein was unaffected.

### Replication of rHPIV3-RSV-F vectors in hamsters and stability of expression of RSV F *in vivo*

**Fig 5:**
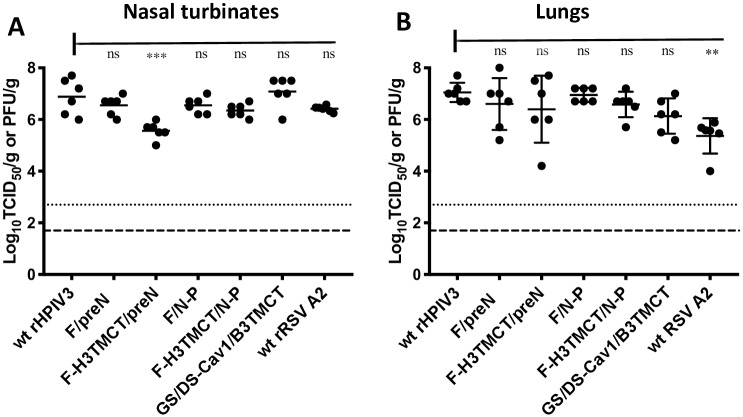
Replication of rHPIV3-RSV-F vectors in hamsters. Groups of 6 hamsters each were inoculated IN with 10^5^ TCID_50_ of each indicated rHPIV3 vector, or 10^6^ PFU of wt RSV. In addition, a previously-described construct, rB/HPIV3/GS/DS-Cav1/B3TMCT (labeled in the Figure as GS/DS-Cav1/B3TMCT) [13, 14], was included for comparison: this consists of the rB/HPIV3 vector expressing, from the second gene position, RSV F with the same codon-optimization and the same HEK, DS, and Cav1 mutations as in the present study, but with the TMCT domains from BPIV3 F protein. At day 5 post-inoculation, virus replication in the nasal turbinates (A) and lungs (B) was assessed by virus titration of tissue homogenates by TCID_50_ limiting dilution assay on LLC-MK2 cells (rHPIV3 and rB/HPIV3 vectors) or immunoplaque assay on Vero cells (wt rRSV). The virus titers for the individual hamsters are plotted as filled circles with the group mean titer indicated by a horizontal line. The limits of detection were 2.2 log_10_TCID_50_/gram for the rHPIV3 and rB/HPIV3 vectors and 1.7 log_10_ PFU/gram for RSV (indicated by dotted and dashed lines, respectively). The statistical significance of the difference between the empty vector and the other viruses was determined by one-way analysis of variance with Tukey’s multiple-comparisons post-test and is indicated by *** (P<0.001) and ns (not significant; P>0.05).

To evaluate the replication efficiency of the rHPIV3-RSV-F vectors in hamsters, groups of 6 hamsters each were inoculated intranasally (IN) with 10^5^ TCID_50_ of wt rHPIV3 or rHPIV3-RSV-F vectors, or with 10^6^ PFU of wt RSV. For comparison, an additional group received 10^5^ TCID_50_ of the construct rB/HPIV3/GS/DS-Cav1/B3TMCT (labeled in Fig 5 as GS/DS-Cav1/B3TMCT), which had been constructed and evaluated in a previous study [14]. This construct consists of the chimeric rB/HPIV3 vector encoding, from the second gene position, a modified RSV F-TMCT protein that is identical to that in the present study except that its TMCT domain was from the F protein of BPIV3 rather than rHPIV3 in order to be compatible with the rB/HPIV3 vector. Nasal turbinates and lungs were collected on day 5 post-inoculation and virus titers in tissue homogenates were determined by TCID_50_ assay on LLC-MK2 cells (rHPIV3 and rB/HPIV3 vectors) or immunoplaque assay on Vero cells (RSV) (Fig 5). All viruses replicated at levels similar to wt rHPIV3 in both the lungs and nasal turbinates except F-H3TMCT/preN, which had a modestly but significantly reduced titer in the nasal turbinates but not the lungs (Fig 5A and 5B). The replication of wt RSV in the nasal turbinates was similar to the other rHPIV3-RSV-F vectors but was reduced in the lungs (Fig 5A and 5B).

**Fig 6:**
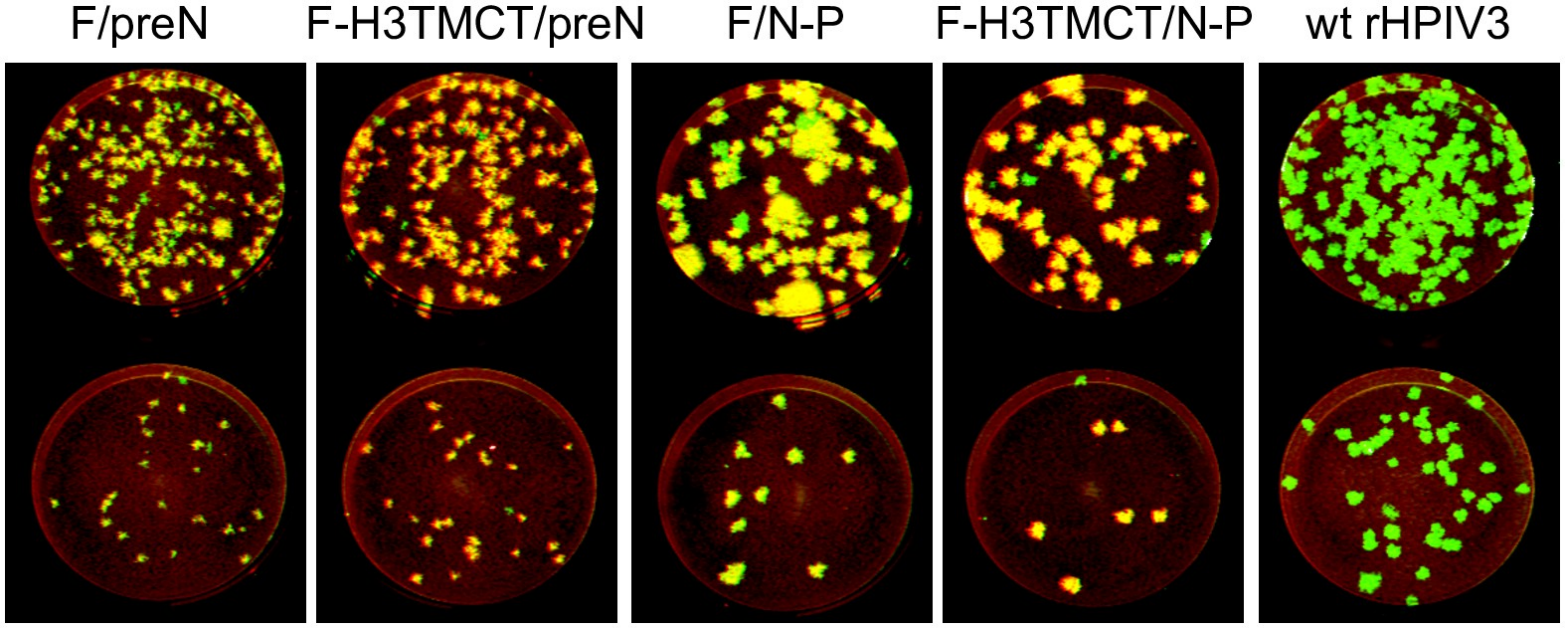
Stability of expression of RSV F protein by rHPIV3-RSV-F vectors after replication in the hamster respiratory tract. Nasal turbinate and lung homogenates prepared as part of the experiment described for Fig 5 were assayed for expression of RSV F protein by a double-staining immunofluorescence plaque assay as described for Fig 2. Representative stained plaques for two dilutions of a lung sample are shown for each group. Quantification is shown in Table 2.

To determine the stability of expression of RSV F protein by the rHPIV3 vectors after 5 days of replication *in vivo*, nasal turbinate and lung homogenates from the experiment in Fig 5 were analyzed by the double-staining immunofluorescence plaque assay (Fig 6), and the percentage of PFUs expressing RSV F detected by the mixture of three RSV F MAbs was determined (Table 2). For each of the four rHPIV3 vectors, RSV F was detected for >93% of PFUs isolated from both tissues (Table 2), as indicated by yellow plaques (examples of virus from hamster lung homogenates are shown in Fig 6). This approached the high percentage of expression observed for the rB/HPIV3/GS/DS-Cav1/B3TMCT construct evaluated in parallel (Table 2). Green plaques, indicating loss of detectable expression of RSV F protein, were slightly more abundant for the F/preN and F/N-P constructs compared to their H3TMCT derivatives.

**Table 2:** Stability of expression of RSV F protein by rHPIV3 and rB/HPIV3 vectors following replication in the respiratory tract of hamsters.

Virus	Hamster#	Nasal turbinates	Lungs
F/preN	1435	98	98
	1436	98	95
	1437	98	99
	1438	100	99
	1439	99	96
	1440	99	93
F-H3TMCT/preN	1441	98	99
	1442	99	96
	1443	98	98
	1444	99	97
	1445	100	98
	1446	96	97
F/N-P	1447	94	99
	1448	94	97
	1449	94	100
	1450	99	95
	1451	99	97
	1452	97	94
F-H3TMCT/N-P	1453	98	93
	1454	98	98
	1455	98	93
	1456	98	97
	1457	98	100
	1458	98	97
B/HPIV3 GS/DS-Cav1/B3TMCT	1459	100	100
	1460	99	100
	1461	100	100
	1462	100	94
	1463	94	99
	1464	100	100

*Six-week old hamsters were inoculated IN with 10^5^ TCID_50_ of the indicated viruses in a 100 μL volume. At 5 days post-infection, nasal turbinates and lungs were collected and tissue homogenates were analyzed by a double-staining immunofluorescence plaque assay on Vero cells to assess the stability of RSV F expression. The percentages of PFUs expressing RSV F, detectable by the conformationally-dependent anti-F MAbs, in the nasal turbinate and lung samples from each hamster are shown.

### Serum RSV- and HPIV3-neutralizing antibody responses

**Fig 7:**
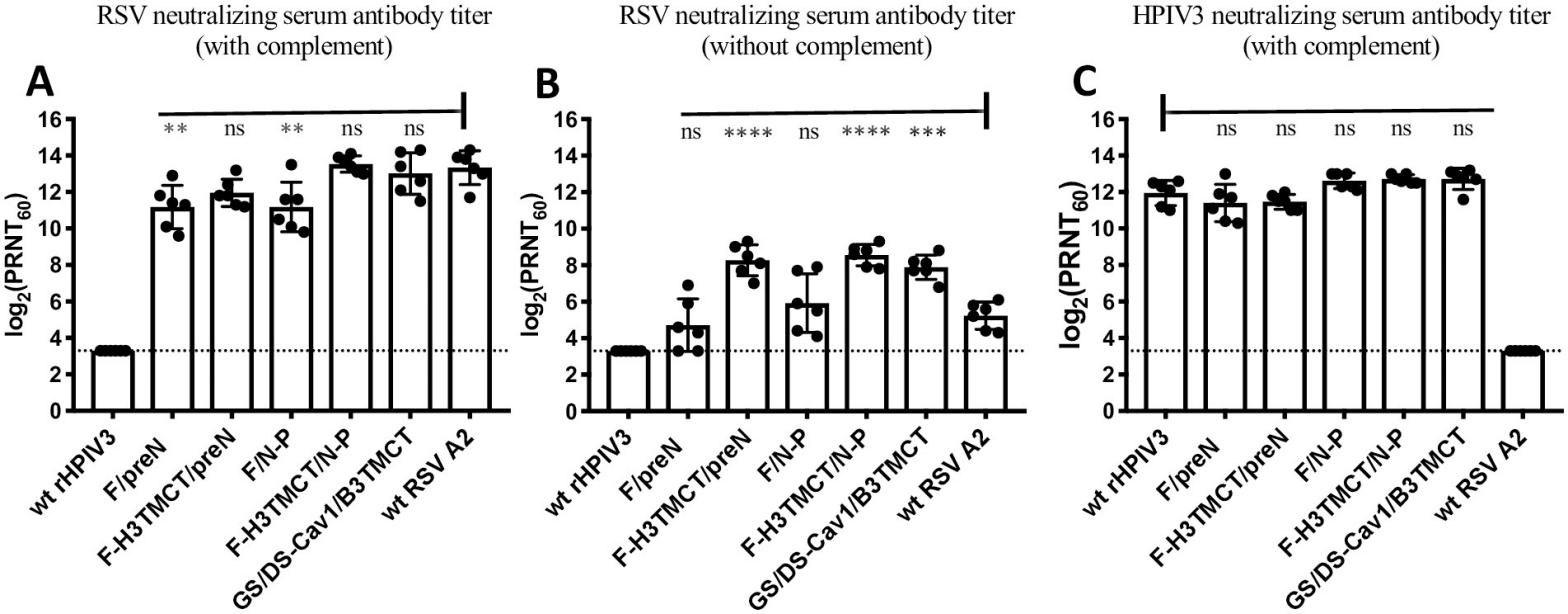
Serum RSV- and HPIV3-neutralizing antibody responses in hamsters following intranasal inoculation with rHPIV3-RSV-F vectors and wt RSV. Additional hamsters in groups of 6 were inoculated as described in the legend to Fig 5 with the indicated HPIV3-RSV-F vectors, or the B/HPIV3 vector GS/DS-Cav1/B3TMCT, or wt RSV. (A, B) Serum RSV-neutralizing antibody titers. Sera were collected at day 28 post-immunization and assayed for RSV-neutralizing antibodies in the presence (A) or absence (B) of added guinea pig complement. (C) Serum HPIV3-neutralizing antibody titers. The 28-day sera were assayed for HPIV3-neutralizing antibodies in the presence of added complement. The results of A-C are presented as log_2_ 60% plaque reduction neutralization test (PRNT_60_) titers for each hamster (filled circles), the mean titer of each group is shown as a bar with vertical lines indicating the standard error of the mean. The statistical significance of the differences between the rHPIV3 and B/HPIV3 vectors versus wt RSV (A and B) or versus wt rHPIV3 empty vector (C) was determined by one-way analysis of variance with Tukey’s multiple-comparisons post-test and is indicated by ** (P<0.01), *** (P<0.001), **** (P<0.0001), and ns (not significant, P>0.05).

To assess the induction of serum virus-neutralizing antibodies, hamsters were inoculated in the same manner as described above for Fig 5 and, on day 28 post-inoculation, sera were collected. These specimens were analyzed for RSV- and HPIV3-neutralizing antibody titers by 60% plaque reduction virus neutralization assays, using rRSV and rHPIV3 viruses that express GFP, with added complement (RSV and HPIV3) or without complement (RSV) (Fig 7). The assay performed with complement is thought to detect a broader array of RSV-specific antibodies, whereas the assay performed without complement would detect only those antibodies that can directly neutralize RSV and are considered to be “high quality”. Using the assay with complement, rHPIV3-RSV F vectors were found to induce high titers of RSV-neutralizing antibodies (Fig 7A). The titers induced by F-H3TMCT/preN and F-H3TMCT/N-P were comparable to those of wt RSV, whereas those for F/preN and F/N-P were significantly lower. The assay without added complement showed the titers of high quality RSV-neutralizing antibodies for the F/preN and F/N-P viruses to be similar to those of wt RSV, whereas those for the F-H3TMCT/preN and F-H3TMCT/N-P viruses were significantly higher (~8-fold) (Fig 7B). All of the vectors that expressed RSV F stimulated HPIV3-neutralizing antibodies equally well and similar to the empty wt rHPIV3 backbone (Fig 7C), suggesting that the RSV F insert had no detectable effect on rHPIV3 immunogenicity.

### rHPIV3-RSV-F vectors protect hamsters against wt RSV challenge

**Fig 8:**
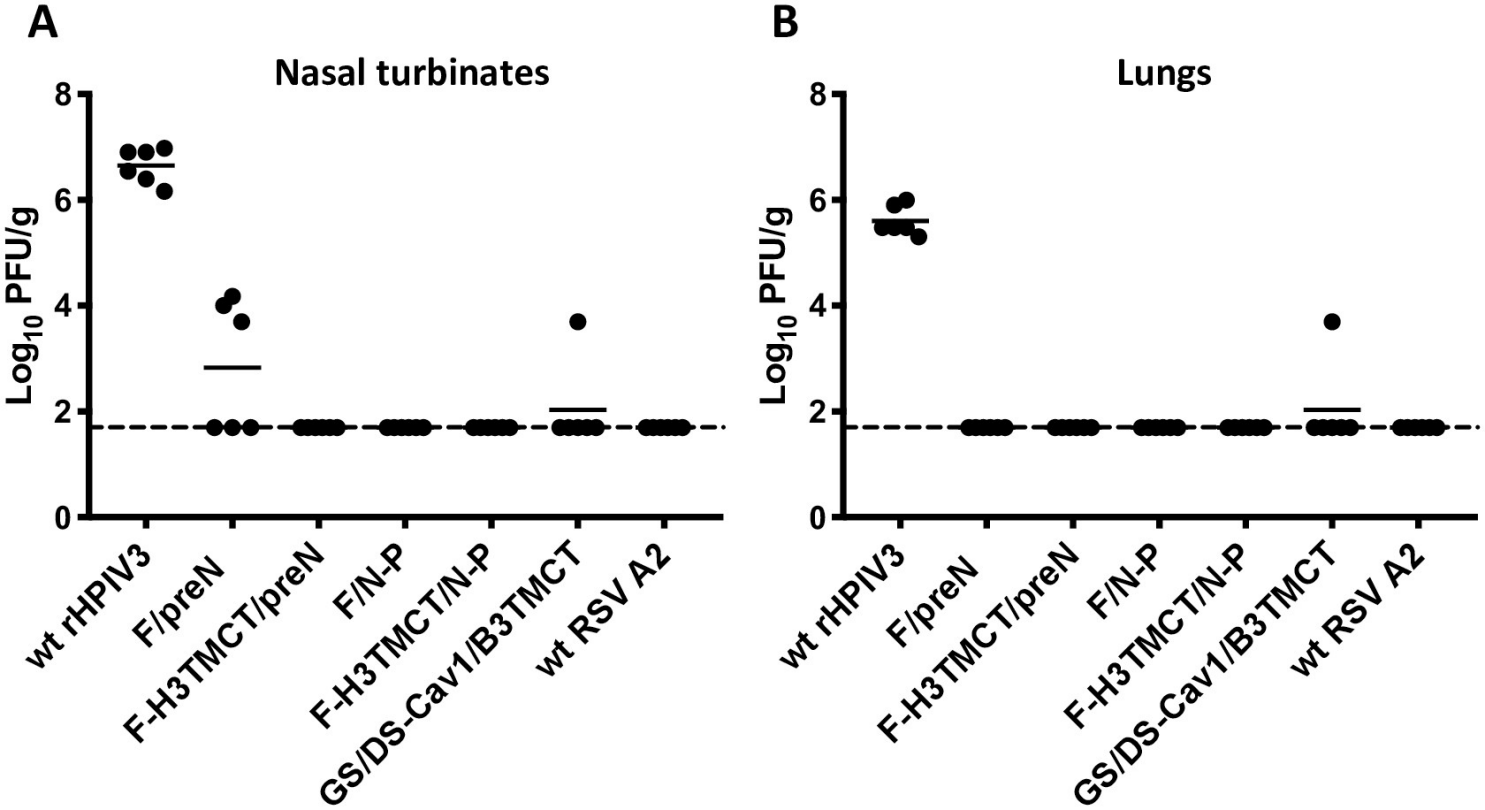
Protection of hamsters against wt RSV challenge. Hamsters immunized with the indicated viruses in the experiment described for Fig 7 were challenged IN with 10^6^ PFU of wt RSV at day 30 post-immunization. The animals were sacrificed on day 3 post-challenge, and the titers of challenge virus in the nasal turbinates (A) and lungs (B) were determined by immunoplaque assay. The RSV titer is shown for each hamster (filled circles) with the group mean titer indicated by a horizontal line. Dashed line indicates the assay detection limit.

The ability of vaccine candidates to protect against RSV infection was assessed by IN challenge with wt RSV. The hamsters that had been immunized to evaluate serum antibody responses in the previous section were challenged IN on day 30 post-immunization with 10^6^ PFU of wt RSV. Three days post-challenge, the animals were sacrificed and nasal turbinates and lungs were harvested, prepared as tissue homogenates, and assayed by immunoplaque assay to determine challenge RSV titers (Fig 8A and 8B). Immunization with F-H3TMCT/preN, F/N-P, and F-H3TMCT/N-P conferred complete protection in both the upper and lower respiratory tract, with no detectable virus replication, and the same results were obtained with wt RSV. F/preN provided complete protection in the lungs but not in the nasal turbinates, where a low level of RSV replication was detected in 3 of 6 hamsters, although at much lower titers than observed for the empty wt rHPIV3 vector negative control. The rB/HPIV3/DS-Cav1/B3TMCT virus [14] included as a reference provided nearly-complete protection against RSV challenge.

### Stability of RSV F expression during serial passage in Vero cells

**Fig 9:**
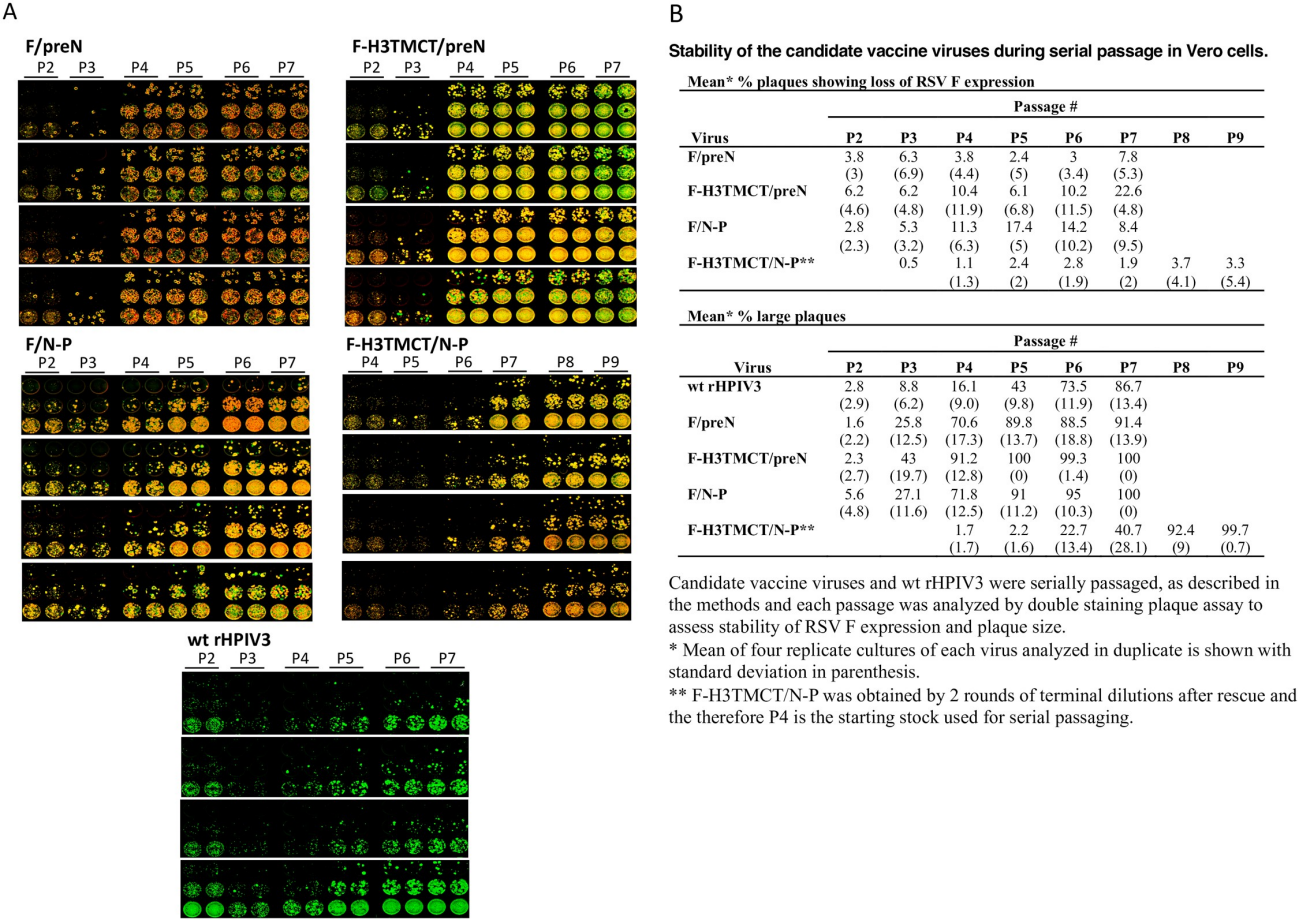
Serial passage of HPIV3-RSV F vectors in Vero cells: Analysis of the stability of RSV F expression. The P4 stock of F-H3TMCT/N-P, P2 stock of F/preN, F-H3TMCT/preN, and F/N-P, or wt rHPIV3 was passaged serially five times in Vero cells, with four replicates of each. Virus samples from each passage were analyzed by double-staining plaque assay on Vero cells to measure the stability of RSV F expression. A. Scans of the immunostained monolayers made with the Odyssey infrared imaging system (Licor) using 800-nm and 680-nm channels: HPIV3 plaques expressing RSV appear as yellow, while HPIV3 plaques in which expression of RSV F was silenced appear as green. Large plaque variants also are evident in some wells. **B**. Green plaques indicating loss of RSV F expression, and large plaque variants, were counted and are shown as mean % plaques with loss of RSV F expression and mean % large plaques.

The stability of the four rHPIV3-RSV-F vectors was evaluated by serial passage of the terminally diluted P4 stock of F-H3TMCT/N-P and the P2 stocks of rest of the three constructs (in four replicate passages per construct) for five additional passages followed by evaluation using double-staining immunofluorescence plaque assay. Serial passage resulted in a gradual loss of RSV F expression, with the following order of increasing mean loss of expression at P7: F-H3TMCT/N-P (1.9%), F/preN (7.8%), F/N-P (8.4%); and F-H3TMCT/preN (22.6%) (Fig 9A and 9B). Thus, the most unstable construct had both H3TMCT and preN, but these features also were present individually in the two constructs with the least loss of expression. In addition, the level of loss of RSV F expression among the 4 replicates of each construct at each passage level had large values of standard deviation (Fig 9A and 9B), indicative of considerable variability.

Serial passage also resulted in an increase in the number of large-size plaque variants for all viruses. This was also evident for the empty wt rHPIV3 backbone, suggesting that the large-plaque phenotype was a characteristic of the HPIV3 backbone rather than the RSV F insert. The majority plaques was of the large variety by P4 for all viruses except F-H3TMCT/N-P and wt rHPIV3, for which this occurred at P8 and P6, respectively. The appearance of large plaques was consistent with our previous report [12] where vector mutations in HN, F, and L were observed. Since HN mutations, particularly H552Q, were consistently detected in the previous study for viruses forming large plaques, in the present study we sequenced the HN encoding region for 2 replicates of each passage for all four vaccine candidates and the empty wt rHPIV3 vector. No mutations were detected in HN, suggesting mutations may be present elsewhere such as in F and/or L as previously described [12].

## Discussion

We evaluated rHPIV3 JS strain as a vaccine vector to express modified forms of the RSV F protein from an added gene inserted at two different locations in the vector genome. A rHPIV3 vector expressing an RSV protective antigen offers a number of advantages compared to an attenuated RSV strain. It provides a bivalent live vaccine against the two most important viral agents of pediatric respiratory illness worldwide. Also, the HPIVs replicate *in vitro* to higher titers than RSV and are physically more stable, which would facilitate manufacture and distribution and would be especially important for use in the developing world where most RSV mortality occurs. Because the supernumerary RSV F protein is not needed for vector replication, it can be engineered with mutations that stabilize it in the pre-F conformation (e.g., DS-Cav1), and also can be engineered (TMCT modification) to be packaged in the vector particle for increased immunogenicity (Introduction). We also modified the RSV F insert by codon optimization and by introducing amino acid changes that make the encoded RSV F protein identical to the early-passage clinical isolate of strain A2. HPIV-vectored vaccines have the potential for use as a primary vaccine or as a booster following a primary immunization with a live-attenuated RSV vaccine or an HPIV vector based on a heterologous serotype (e.g., HPIV1 versus HPIV3).

As noted in the Introduction, we have been developing the attenuated recombinant chimeric rB/HPIV3 virus as a vector to express the RSV F or G protein from an added gene, and evaluated a number of strategies described above to modify RSV F and G for improved immunogenicity [12–15, 23]. In addition, rB/HPIV3 expressing unmodified RSV F also was previously evaluated by MedImmune in seronegative young children in a phase I clinical study [11]. In that study, the construct appeared to be well-tolerated and was moderately immunogenic against HPIV3 but poorly immunogenic against RSV. That vaccine lot, as well as vaccine shed from vaccinees, exhibited substantial frequencies of loss of expression of RSV F, which might account for the poor RSV immunogenicity. However, we recently showed that loss of expression of RSV F by a PIV3 or PIV1 vector can be minimized by monitoring virus lots with a double-immunostaining plaque assay such as described in the present study [12–16]. We also recently showed that the immunogenicity of the RSV F protein expressed by rB/HPIV3 can be substantially increased by strategies described above, especially pre-F stabilization and TMCT-mediated packaging into the vector particle [12–15].

However, there are two potential deficiencies in the rB/HPIV3 vector. First, this chimeric virus contains only the F and HN proteins from HPIV3. The remaining proteins (i.e., N, P, M, and L) are “internal” proteins from BPIV3 and share 84%, 58%, 89%, and 89% amino acid sequence identity, respectively, with HPIV3. In previous studies, the expression of “internal” proteins from a PIV induced substantial protection against challenge that likely was mediated by cellular immunity [24]. Thus, there likely would be an advantage to protection against HPIV3 infection in having immunity against the full complement of HPIV3 proteins rather than F and HN from HPIV3 and the rest from BPIV3. Second, we previously showed that the DS-Cav1 and TMCT modifications conferred substantial additional attenuation to the rB/HPIV3-RSV-F constructs in hamsters and non-human primates, compared to the empty rB/HPIV3 vector or rB/HPIV3 expressing unmodified RSV F. Since the rB/HPIV3 vector, on its own or expressing unmodified RSV F protein, was well tolerated in infants and young children, any additional attenuation might make the construct over-attenuated in humans such that immunogenicity might be suboptimal. That can only be determined in future clinical studies. For these reasons, in the present study we began the development of rHPIV3 JS as vector so that it will be available should rB/HPIV3-RSV-F prove over-attenuated.

The HPIV3 JS strain ostensibly is a wild type virus, although previous clinical evaluation in adults showed that it was highly restricted, avirulent, and poorly immunogenic, even when the adult subjects had been selected for low serum HPIV3-specific antibodies [25]. Therefore, the JS strain may naturally be somewhat attenuated in humans. The goal of the present study was to evaluate the level of attenuation, immunogenicity, protective efficacy, and stability of unmodified rHPIV3 JS strain expressing the DS-Cav1 form of RSV F, with and without TMCT modification. In subsequent studies, we will add additional attenuating mutations as needed to achieve an optimal balance of attenuation and immunogenicity, using attenuating mutations that have been identified and characterized previously [26–28].

Analysis of the initial transfection series by double-staining immunofluorescence plaque assay showed that each of the virus stocks contained a substantial minority sub-population of plaques that exhibited loss of expression of RSV F. In addition, we were surprised to find that every stock had a second minority sub-population of plaques with a large-plaque phenotype. The large-plaque phenotype appeared to be unrelated to the expression or loss of expression of RSV F, and also was observed with unmodified rHPIV3 JS strain. In an effort to obtain viral stocks in which these sub-populations were reduced, we performed two rounds of terminal dilution enrichment. However, only in the case of one construct (F-H3TMCT/N-P) did this succeed in providing a P4 stock with low levels of these sub-populations. Therefore, two additional transfections were performed. In contrast with the first transfection series, the stocks from these additional transfections had low backgrounds of large plaques and low loss of expression of RSV F, and thus provided suitable P2 stocks for the remaining three constructs. This showed that the occurrence of these sub-populations was variable.

Additional terminal dilutions of the first transfection series were performed to obtain a number of vector stocks enriched for PFU that had lost expression of RSV F. Sequence analysis of several stocks revealed the presence within the RSV F ORF of translational stop codons or a frameshift leading to premature termination. There also were instances of missense mutations associated with loss of detectable expression of RSV F. These missense mutations may have affected the processing or conformation or epitopes of RSV F, resulting in loss of binding by the three conformationally-dependent MAbs used in the immunofluorescence assay. In previous studies with rB/HPIV3 expressing RSV F from the N-P position, loss of expression of RSV F frequently was associated with mutations in the GE signal of the preceding N gene, which result in readthrough transcription to yield a bicistronic mRNA in which the RSV F ORF was the second ORF [12, 29]. In that position, the RSV F ORF would be translated very inefficiently. However, this type of mutation—involving the upstream GE signal—was not observed in the F/N-P constructs in the present study, for reasons unknown.

We investigated the nature of the large-plaque variants by using terminal dilution to obtain a stock of F-H3TMCT/preN that was enriched for large-plaque variants. Complete genome sequence analysis identified a Y3H mutation in HN and V242G, N248K, C252W, F277V, and I279M mutations in L. Which of these mutations might be responsible for the large-plaque phenotype remains unknown: it will be important to sequence other large-plaque isolates as well as to reintroduce these mutations back into rHPIV3 to assess effects on phenotype. Complete genome sequence analysis of a terminally-diluted large-plaque variant of F/N-P failed to detect any prominent mutations: it may be that multiple minor mutations were involved. Future analysis will include more-thorough biological cloning of large-plaque variants and deep-sequencing analysis linked to long-range RT-PCR.

In addition, we examined the stability of expression of RSV F by subjecting the P4 stock of F-H3TMCT/N-P and the P2 stocks of the remaining three constructs to five additional serial passages (P5-P9 and P3-P7, respectively, with four replicate cultures per construct) in Vero cells, which is the substrate that would be used for vaccine manufacture. Each passage level was analyzed by double-staining immunoplaque assay. The different constructs varied in the loss of RSV F expression, with the order of increasing mean loss of expression at P7 as follows: F-H3TMCT/N-P (1.9%); F/preN (7.8%); F/N-P (8.4%); and F-H3TMCT/preN (22.6%). The proportion of plaques with loss of expression of RSV F gradually increased or sometimes fluctuated with increasing passage level, suggesting that there was not a strong selective advantage to silencing expression. P3 would be the passage level corresponding to clinical trial material, and the loss of F expression at this passage ranged from 0.5% to 6.3%. In the hamster experiment, viruses isolated from the respiratory tract of immunized animals exhibited a high level of RSV F expression (>93% in all nasal turbinate and lung samples) suggesting that there was no evident selective advantage to loss of expression *in vivo*. The loss of expression of RSV F *in vitro* or *in vivo* was not consistently associated with either the TMCT modification or the preN position.

When the stocks of the four constructs were subjected to five additional passages in parallel with wt rHPIV3, as described above, large-plaque viruses were found to constitute >50% of the population by P4 for three of the five viruses, and 22.7% to 100% of the population for all five viruses by P6. When hamsters were inoculated with the P4 stock of F-H3TMCT/N-P and P2 stocks of the other three constructs, which all had minor populations of large-plaque variants, the virus recovered from nasal turbinates and lungs five days later were largely free of large-plaque variants, except for the F/N-P construct that retained a consistent minor population that formed larger sized plaques but had intact expression of RSV F. The observation that these large-plaque variants remained very small sub-populations indicates that they did not appear to have a selective growth advantage in the hamster respiratory tract.

In previous studies by others [30] large-plaque variants of HPIV3 were isolated following passage *in vitro* in the presence of a neuraminidase inhibitor, and were shown to be associated with missense mutations in the HN protein—notably H552Q- that conferred a higher avidity for sialic acid-containing receptor and mediated increased triggering of the HPIV3 F protein fusion activity [31]. These large-plaque viruses were attenuated *in vivo*. Our laboratory also previously generated large-plaque variants of rB/HPIV3-RSV-F during passage in Vero cells [12]. In that case, the large-plaque phenotype was associated with a varying array of missense mutations in HN that mostly were located at the predicted dimer interface. These mutations appeared to be the consequence of two missense markers (T263I and P370T) that had purposefully been added to the HN gene of the original rHPIV3 JS antigenomic cDNA [21] in order to ablate an epitope for a monoclonal antibody with neutralization, hemagglutinin-inhibition, and neuraminidase-inhibition activities: when these two markers were restored to their wild type assignments, the appearance of large-plaque variants was greatly reduced. Thus, large-plaque variants of HPIV3 have been previously observed that appeared to involve increasing the efficiency of the HN attachment activity, but it is not known whether large-plaque variants can arise for other reasons.

It also is not known whether the large plaque phenotype is unique to the JS strain and its chimeras or is a general property of HPIV3 –we have not observed it with HPIV1. In our laboratory, HPIV3 titration historically has been done mostly by limiting dilution assay, and only in recent years have we routinely been using plaque assays, which revealed the large plaques. The observation that the large plaque variants appeared to decrease rather than increase during viral replication *in vivo* suggests that they are neutral or attenuating and probably will be acceptable as a minority population in vaccine material once the basis for this phenotype is better understood. Overall, F-H3TMCT/N-P was the most stable of the four candidates, maintaining expression of RSV F even after P9 and showing a low percentage of large plaque variants through P6. From these data we infer that the number of passages to manufacture the vaccine lot must be kept to a minimum and stringent quality control must be in place to detect insert and backbone instability.

Full-length RSV F (present in constructs F/preN and F/N-P) was packaged very inefficiently into the rHPIV3 vector particle, as observed previously with rB/HPIV3 and rHPIV1 vectors [13, 14]. Substitution of the RSV F TMCT domain with that of the HPIV3 vector F protein (constructs F-H3TMCT/preN and F-H3TMCT/N-P) substantially increased its packaging in the particle such that it was packaged, per microgram of virion protein, at an efficiency similar to that of wt RSV. This was associated with a corresponding decrease in packaging of the rHPIV3 F protein, presumably due to competition between these proteins due to the common TMCT region specific to HPIV3 F protein. Surprisingly, this did not affect virus replication *in vitro*, as the TMCT versions replicated with efficiencies similar to their counterparts without the H3TMCT modification. There also was no apparent association between the TMCT modification and the loss of expression of RSV F, or the large-plaque variant.

Multistep growth curves indicated that the insertion of RSV F at the first position in front of the vector N gene (F/preN and F-H3TMCT/preN) reduced the kinetics of replication in vitro, whereas insertion between N and P (F/N-P and F-H3TMCT/N-P) had little effect. The presence of the TMCT mutation was not inhibitory at either position. However, constructs with RSV F at the first position did reach final titers similar to other vectors and the empty rHPIV3 vector by day 7 post-infection. Reduced replication of F/preN and F-H3TMCT/preN also was suggested by their smaller plaque sizes. Analysis of intracellular protein expression revealed reduced expression of vector N, P, F, and HN proteins by the F/preN and F-H3TMCT/preN constructs, as compared to the F/N-P and F-H3TMCT/N-P constructs and the empty rHPIV3 vector. The reduced expression of vector proteins associated with insertion into the first gene position likely contributes to the reduced replication observed early during infection.

The level of expression of RSV F protein by the vectors in LLC-MK2 cells could not be compared to that of wt RSV because of the poor wt RSV replication in this cell line, the reason for which is unknown: the RSV F_0_ protein was observed to be very poorly cleaved in these cells compared to Vero cells, but this would not be expected to affect a single-step infection. In Vero cells, where both the rHPIV3 vectors and wt RSV replicated well, expression of RSV F by the F/preN, F-H3TMCT/preN, and F/N-P vectors was somewhat less compared to wt RSV, whereas expression by F-H3TMCT/N-P was significantly greater. Interestingly, the F-H3TMCT/preN and F-H3TMCT/N-P viruses had a higher level of expression of RSV F compared to their counterparts without the TMCT modification. This was particularly evident with the F-H3TMCT/N-P construct. The basis for this higher level of expression is unknown. One possibility is that the vector-specific TMCT domain may have stabilized the RSV F protein due to interactions with other rHPIV3 proteins.

All of the vector constructs replicated efficiently in the nasal turbinates and lungs of hamsters. The F-H3TMCT/preN construct alone exhibited modest but significant restriction in the nasal turbinates—but not the lungs—compared to the other vector constructs and wt rHPIV3. When sera collected 28 days following vaccination were evaluated for RSV-neutralizing activity in the presence of added complement, the titers were either similar to (F-H3TMCT/preN and F-H3TMCT/N-P) or lower than (F/preN and F/N-P) that for wt RSV. *In vitro* neutralization also was evaluated without added complement in order to measure high-quality antibodies capable of directly neutralizing RSV. The highest antibody titers were observed for F-H3TMCT/preN and F-H3TMCT/N-P: these were significantly higher than for the F/preN and F/N-P as well as wt RSV, even though wt RSV was administered at a higher dose. F-H3TMCT/preN, F-H3TMCT/N-P, and F/N-P provided complete protection against RSV challenge in the upper and lower respiratory tract. F/preN provided complete protection in the lungs, but only partial protection in the nose: RSV replication was detected in 3 of 6 animals albeit at significantly reduced titers.

Our previous studies with rB/HPIV3 and rHPIV1 vectors showed that expression of the RSV F protein with mutations increasing the stability of the pre-F conformation was associated with increased induction of high-quality RSV-neutralizing antibodies detected in the absence of added complement [13, 14] [16]. In the present study, the addition of the H3TMCT mutation similarly resulted in a significant increase in the induction of these high-quality antibodies. Our future studies would entail the systematic introduction of attenuating mutations into the rHPIV3 vectors, particularly the F-H3TMCT/N-P construct, followed by evaluation of their performance. The present level of large-plaque variants and loss of expression of RSV F expression may be acceptable for vaccine purposes, although we will investigate the mechanisms and conditions involved and work to further reduce the occurrence of these variants. In conclusion, this study evaluated four RSV/HPIV3 bivalent vaccine candidates that demonstrated promising immunogenicity and merit further development as RSV IN vaccines for pediatric use.

## Materials and methods

### Cells and viruses

African green monkey kidney epithelial Vero cells (ATCC CCL-81), rhesus macaque kidney LLC-MK2 epithelial cells (ATCC CCL-7), and baby hamster kidney BHK BSR-T7/5 cells that constitutively express the T7 RNA polymerase were maintained as previously described [16]. Recombinantly-derived wt RSV strain A2 (GenBank accession number KT992094) and a derivative expressing green fluorescent protein were previously described [32]. wt rHPIV3 was a recombinant version of strain JS (GenBank accession number Z11575) modified as described below. rB/HPIV3 expressing the DS-Cav1/B3TMCT version of RSV F has been previously described [14].

### Design of rHPIV3 vectors expressing RSV pre-F with or without H3TMCT

In order to express the RSV F protein from the first (pre-N) or second (N-P) gene position of rHPIV3 (Fig 1), two modifications were made in the rHPIV3 genome. First, either a unique *Blp*I (pre-N) or a unique *Asc*I (N-P) site was introduced in the antigenome cDNA at nt position 103–109 (TTTCAAA mutated to GCTTAGC) or 1675–1682 (ATCTAAAT mutated to GGCGCGCC), respectively, by site directed mutagenesis without affecting the viral genome length (Fig 1). The *Blp*I and *Asc*I sites are located in the upstream and downstream non-coding regions of the N gene, respectively, and each vector had one or the other site, not both. Second, two HN mutations T263I and P370T that had previously been introduced as markers in the rHPIV3 JS reverse genetics system [21] were corrected to wt assignments T263 and P370, respectively.

cDNA encoding the RSV F protein of strain A2 (GenBank accession number KT992094) was designed to be codon-optimized (Genscript, Piscataway NJ) (GenBank accession # MN746317) for human expression. Two amino acid substitutions K66E and Q101P (called the HEK mutations) were included to make the amino acid sequence identical to that of an early passage of strain A2, and which also increase expression [15]. In addition, the DS (S155C and S290C) and Cav1 (S190F and V207L) mutations were included that increase the stability of the pre-F conformation. A second version of this cDNA was designed in which the TMCT domain of RSV F protein (residues 530–574; Fig 1C) was replaced with that of HPIV3 F (H3TMCT, residues 494–539; Fig 1C) to enable efficient incorporation in the vector virions. The locations of the RSV F and HPIV3 F TMCT domains were previously described [13, 33]. These two cDNAs, encoding full-length RSV F protein or the RSV F-H3TMCT protein, were further designed to be flanked by HPIV3 gene-start (GS) and gene-end (GE) transcription signals, an intervening intergenic (IG) sequence, and either a set of *Blp*I or *Asc*I sites, as shown in Fig 1A and 1B. This resulted in four RSV F cDNAs, namely full-length RSV F and RSV-F-H3TMCT with *Blp*I sites (Fig 1A) or *Asc*I sites (Fig 1B). These four cDNAs were synthesized commercially (Genscript). The pair of RSV F cDNAs with *Blp*I sites were inserted individually at the first (pre-N) gene position of rHPIV3 (Fig 1A), to yield the constructs F/preN and F-H3TMCT/preN, and the pair with *Asc*I sites was inserted individually at the second (N-P) gene position of HPIV3 to yield the constructs F/N-P and F-H3TMCT/N-P. All constructs were designed to maintain a hexameric genome length and preserve hexamer phasing of the vector genes. The RSV F inserts at the pre-N and N-P position had the hexamer phasing of the N and P genes, respectively. The vector constructs were recovered in duplicate by co-transfecting BHK BSR T7/5 cells with the anti-genome plasmids and three expression plasmids expressing the HPIV3 N, P, and L proteins (see Results).

### Double-staining immunofluorescense plaque assay

As described previously [15], Vero cells in 24-well plates were infected with 10-fold serially diluted virus and incubated at 32°C overlaid with Opti-MEM I medium containing 0.8% methylcellulose, 2% fetal bovine serum (FBS), and 1x L-glutamine. At 6 days post-infection, cells were fixed twice with ice-cold 80% methanol. Plaques were immunostained with a rabbit polyclonal antiserum raised against HPIV3 virions [34] and a mixture of three conformation-dependent murine MAbs (1129, 1269, and 1243) to the RSV F protein [22]. The images were acquired using an Odyssey infrared imaging system and plaques were pseudo-colored to appear green and red for rHPIV3 and RSV F, respectively. Plaques co-expressing rHPIV3 and RSV F antigens appear yellow, and those with loss of RSV F expression (or with an alteration in F conformation sufficient to prevent binding by the conformation-dependent MAbs) appear green.

### Multi-cycle replication curve of rHPIV3 vectors in cell culture

Vero cells in 6-well plates were infected in triplicate with rHPIV3 vectors at a multiplicity of infection (MOI) of 0.01 TCID_50_ per cell. After virus adsorption, the inoculum was removed, cells were washed, and 3 ml of medium was added to each well followed by incubation at 32C for a total of 7 days. At 24 h intervals, 0.5 ml of culture medium was collected and flash-frozen, and 0.5 ml of fresh medium was added to each well. The collected samples were titrated together in LLC-MK2 cells in 96-well plates by the TCID_50_ limiting dilution method using hemadsorption with guinea pig erythrocytes to identify the infected wells.

### Western blot analysis

LLC-MK2 or Vero cells in 6-well plates were infected with rHPIV3 or rHPIV3-RSV F at an MOI of 3 TCID_50_ per cell or wt RSV at an MOI of 3 PFU per cell. After incubation at 32C for 48 h, cells were washed once with cold PBS and lysed with 300 ul 1X LDS lysis buffer (Thermo Fisher Scientific) containing 1X reducing reagent (Thermo Fisher Scientific). Cell lysates were passed through a QIAshredder column (Qiagen, Valencia CA), heated for 5 min at 95°C, and 20 μl of each sample was loaded onto a 4–12% Bis Tris NuPAGE gel (Thermo Fisher Scientific). Proteins were electrophoresed in the presence of antioxidant (Thermo Fisher Scientific) and transferred to PVDF membranes using the iBlot system (Thermo Fisher Scientific). Membranes were blocked with blocking buffer (LiCor, Lincoln NE) for 1 h and probed with primary antibodies in blocking buffer overnight at 4°C, followed by incubation with blocking buffer containing infrared dye-labeled secondary antibodies (LiCor). Blot images were acquired and analyzed using Image Studio software (LiCor). Primary antibodies included (i) a murine anti-RSV F MAb (ab43812, Abcam, Cambridge MA); (ii) rabbit polyclonal hyperimmune serum against sucrose gradient-purified HPIV3 virions, or E. coli-expressed rHPIV3 F ectodomain, or an HPIV3 HN peptide (YWKHTNHGKDAGNELETC); and (iii) mouse anti-tubulin monoclonal antibody (MAb) (ThermoFisher Scientific; catalog number A-11126). Secondary antibodies included donkey anti-rabbit IRDye 800CW and donkey anti-mouse IRDye 680LT (LiCor) that were used as per manufacturer’s instructions.

### Replication in hamsters

Groups (n = 6) of 6-week old female Golden Syrian hamsters (Envigo Laboratories, Frederick MD), pre-screened to be RSV- and HPIV3-seronegative, were inoculated IN with 100 μl of Leibovitz’s L15 medium (Thermo Fisher Scientific) containing 10^5^ TCID_50_ of rHPIV3, or 10^6^ PFU of wt RSV. At day 5 post-inoculation, nasal turbinates and lungs were collected and virus replication was assessed by titration of homogenized samples on LLC-MK2 cells using limiting dilution and hemadsorption assay for rHPIV3 or on Vero cells by immunoplaque assay for RSV.

### Immunogenicity and protection against RSV challenge

In a separate study, hamsters in groups (n = 6) were inoculated as described above and sera were collected on day 28 to measure serum RSV- and HPIV3-neutralizing antibody titers. On day 30, hamsters were challenged IN with 10^6^ PFUs of wt RSV A2 strain. Nasal turbinates and lungs were collected on day 3 post-challenge to assess protection against RSV replication by immunoplaque titration of tissue homogenates on Vero cells. Serum titers of RSV F- or HPIV3-specific neutralizing antibodies were determined by a 60% plaque reduction neutralization test (PRNT_60_) on Vero cells in 24-well plates using rRSV or rHPIV3 expressing green fluorescent protein. Sera were heated at 56°C for 30 min to inactivate serum complement, and 4-fold serial dilutions of each serum in duplicate were mixed with an equal volume of diluted virus either with or without added guinea pig complement (Lonza, Walkersville, MD) [35]. After incubation for 30 min at 37°C, the serum/virus mix was transferred onto Vero cell monolayers, rocked for 2 h at 32°C, overlaid with Opti-MEM I containing 2% FBS, 0.8% methylcellulose, 1x L-glutamine, and 50 μg/ml gentamicin (Thermo Fisher Scientific) and incubated for 6 days at 37°C for RSV or 32°C for rHPIV3. The plates were scanned using a Typhoon scanner (GE Healthcare Life Sciences, Pittsburgh, PA), plaques were counted with ImageJ software (W. S. Rasband, U.S. National Institutes of Health, Bethesda, MD, USA [http://imagej.nih.gov/ij/]), and PRNT_60_ titers were calculated by linear regression analysis.

### Stability of viruses on serial passaging in Vero cells

The stability of RSV F expression by the candidate vaccine viruses was evaluated over multiple *in vitro* passages in Vero cells, the cell type used for preparing clinical trial material. Briefly, Vero cells in T-25 flasks were infected with the P4 stock of F-H3TMCT/N-P, the P2 stocks of the other three constructs, or the P2 stock of wt rHPIV3, four replicates of each, at an MOI of 0.01 TCID_50_/cell. Following incubation at 32C for 3 days, culture supernatant was collected and was used to infect fresh Vero cells (MOI of 0.01 TCID_50_/cell calculated based on the day 3 titer predicted by the growth curve in Fig 3) while the rest was stored at -80C for further analysis. The viruses were serially passaged 5 times. The serial passage samples were analyzed by double-staining plaque assay. Virus genome sequencing covering the HN ORF was also performed to determine the basis of plaque phenotypes.

### Ethics statement

All animal studies were approved by the NIH Institutional Animal Care and Use Committee (IACUC) under the animal study protocol number LID 34E. The National Research Council’s Guide for the care and use of laboratory animals and the Public Health Service Policy on humane care and use of laboratory animals served as the guidelines for the care and use of hamsters in this study.

## Supporting information

S1 Checklist The ARRIVE guidelines checklist. (PDF)

S1 File (PDF)
