# Supplementary material for: Human parainfluenza virus type 3 expressing the respiratory syncytial virus pre-fusion F protein modified for virion packaging yields protective intranasal vaccine candidates
Source: PLoS One. 2020 Feb 11;15(2):e0228572. doi: 10.1371/journal.pone.0228572 (PMC7012412; doi:10.1371/journal.pone.0228572)

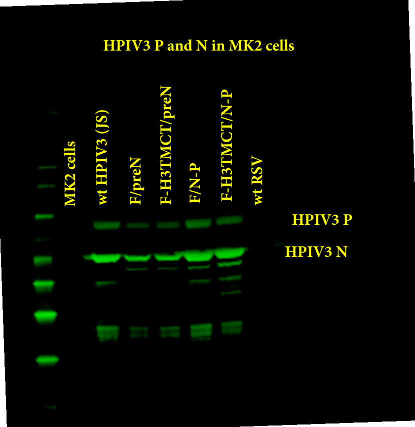
HPIV3 P and N in MK2 cells
MK2 cells
wt HPIV3 (JS)
F/preN
F-H3TMCT/preN
F/N-P
F-H3TMCT/N-P
wt RSV
HPIV3 P
HPIV3 N

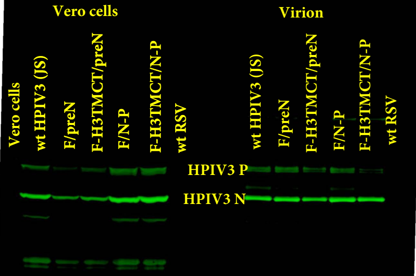

HPIV3 P and N in vero cells and on virion

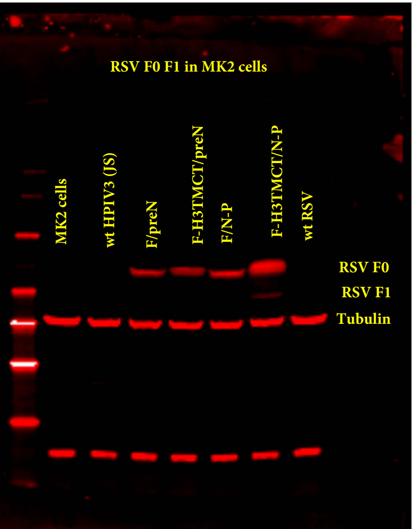

RSV F0 F1 in MK2 cells
MK2 cells
wt HPIV3 (JS)
F/preN
F-H3TMCT/preN
F/N-P
F-H3TMCT/N-P
wt RSV
RSV F0
RSV F1
Tubulin

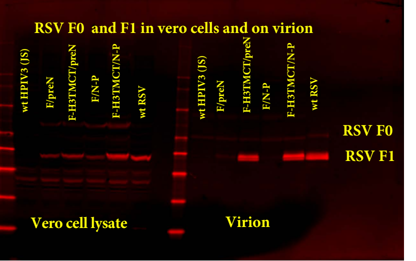
RSV F0 and F1 in vero cells and on virion
wt HPIV3 (JS)
F/preN
F-H3TMCT/preN
F/N-P
F-H3TMCT/N-P
wt RSV
wt HPIV3 (JS)
F/preN
F-H3TMCT/preN
F/N-P
F-H3TMCT/N-P
wt RSV
RSV F0
RSV F1
Vero cell lysate
Virion

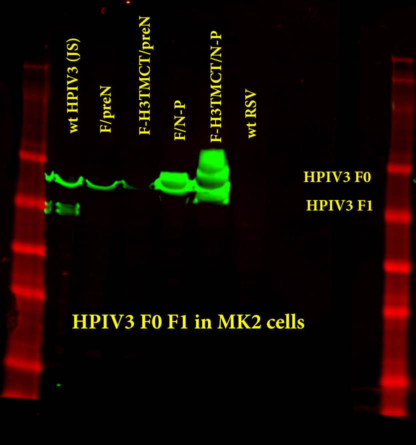
wt HPIV3 (JS)
F/preN
F-H3TMCT/preN
F/N-P
F-H3TMCT/N-P
wt RSV
HPIV3 F0
HPIV3 F1
HPIV3 F0 F1 in MK2 cells

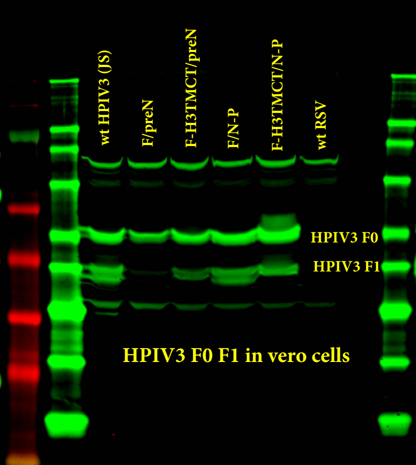
wt HPIV3 (JS)
F/preN
F-H3TMCT/preN
F/N-P
F-H3TMCT/N-P
wt RSV
HPIV3 F0
HPIV3 F1
HPIV3 F0 F1 in vero cells

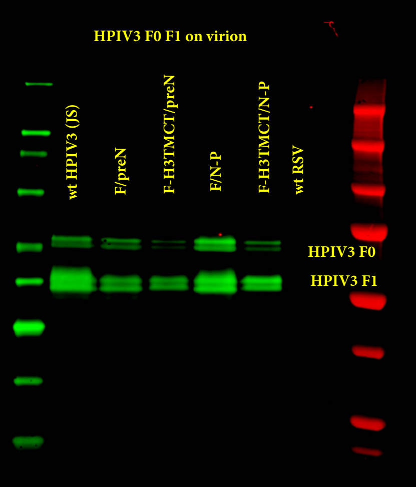
HPIV3 F0 F1 on virion
wt HPIV3 (JS)
F/preN
F-H3TMCT/preN
F/N-P
F-H3TMCT/N-P
wt RSV
HPIV3 F0
HPIV3 F1

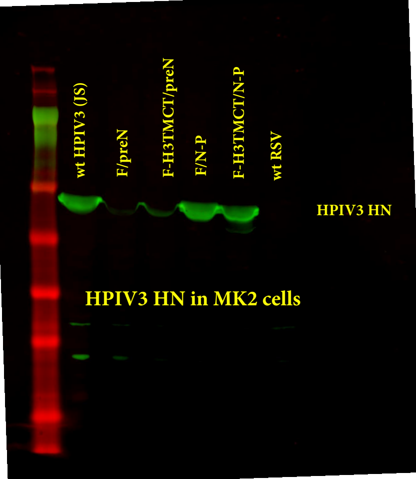

wt HPIV3 (JS)
F/preN
F-H3TMCT/preN
F/N-P
F-H3TMCT/N-P
wt RSV
HPIV3 HN
HPIV3 HN in MK2 cells

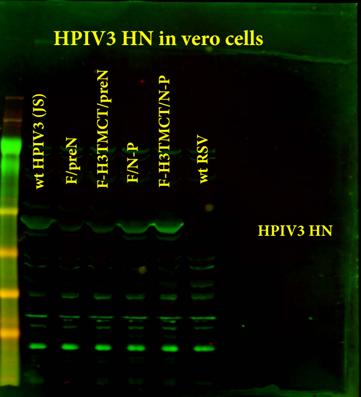
HPIV3 HN in vero cells
wt HPIV3 (JS)
F/preN
F-H3TMCT/preN
F/N-P
F-H3TMCT/N-P
wt RSV
HPIV3 HN

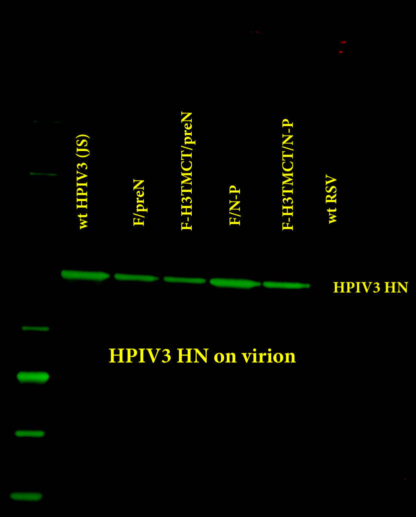
wt HPIV3 (JS)
F/preN
F-H3TMCT/preN
F/N-P
F-H3TMCT/N-P
wt RSV
HPIV3 HN
HPIV3 HN on virion

Tubulin in MK2 and Vero cell lysates

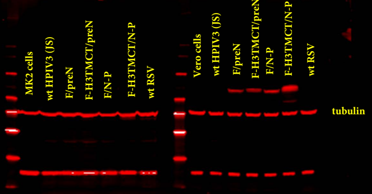

Supplement: S1 File — (PDF) [file pone.0228572.s002.pdf]
